# Mechanistic Exploration of *N*,*N′*-Disubstituted Diamines as Promising Chagas Disease Treatments

**DOI:** 10.3390/ph19010119

**Published:** 2026-01-09

**Authors:** Alejandro I. Recio-Balsells, Chantal Reigada, María Gabriela Mediavilla, Esteban Panozzo-Zénere, Miguel Villarreal Parra, Patricia S. Doyle, Juan C. Engel, Claudio A. Pereira, Julia A. Cricco, Guillermo R. Labadie

**Affiliations:** 1 Instituto de Química Rosario (IQUIR-CONICET-UNR), Suipacha 531, Rosario 2000, Argentina; reciobalsells@iquir-conicet.gov.ar (A.I.R.-B.); estebanpanozzozenere@gmail.com (E.P.-Z.); villarrealparra@iquir-conicet.gov.ar (M.V.P.); 2 Facultad de Medicina, Instituto de Investigaciones Médicas A. Lanari, Universidad de Buenos Aires, Buenos Aires 1113, Argentina; chanreigada@hotmail.com (C.R.);; 3 Laboratorio de Parasitología Molecular, Instituto de Investigaciones Médicas (IDIM), Consejo Nacional de Investigaciones Científicas y Técnicas, Universidad de Buenos Aires, Buenos Aires 1113, Argentina; 4 Instituto de Biología Molecular y Celular de Rosario (IBR-CONICET-UNR), Ocampo y Esmeralda, Rosario 2000, Argentina; mediavilla@ibr-conicet.gov.ar (M.G.M.); cricco@ibr-conicet.gov.ar (J.A.C.); 5 Department of Pathology, Sandler Center for Drug Discovery, University of California San Francisco, San Francisco, CA 94158-2330, USA; patricia.doyle.engel@gmail.com (P.S.D.); juan.engel@ucsd.edu (J.C.E.); 6 Departamento de Química Orgánica, Facultad de Ciencias Bioquímicas y Farmacéuticas, Universidad Nacional de Rosario, Suipacha 531, Rosario 2000, Argentina

**Keywords:** neglected diseases, Chagas disease, amastigote inhibition, polyamine transport, disubstituted diamines

## Abstract

**Introduction:** Chagas disease, caused by the protozoan *Trypanosoma cruzi*, remains a major public health concern due to the limited effectiveness of current treatments, especially in the chronic stage. **Objective:** Here, we wanted to advance a library of 30 *N*,*N′*-disubstituted diamines as promising antichagasic agents and gain insight into the mechanism of action. **Methods:** The library was evaluated for activity against the *T. cruzi* amastigote stage and trypanocidal efficacy. In addition, selected compounds were tested as potential polyamine transport inhibitors, and a fluorescent analog was employed to investigate compound internalization. **Results**: Five compounds exhibited potent activity (pIC_50_ > 6.0), particularly those with short aliphatic linkers (3–6 carbon atoms), suggesting a structure–activity relationship favouring shorter chains. Mechanistic studies showed that compound **3c** strongly inhibited polyamine transport, a vital pathway in *T. cruzi*, though this was not a universal mechanism among active hits, indicating the potential for multiple targets. A fluorescent analog confirmed intracellular uptake in amastigotes but lacked antiparasitic activity, likely due to disrupted pharmacophoric features. Importantly, none of the compounds demonstrated trypanocidal activity in long-term assays, and some showed cytotoxicity, particularly in the benzyloxy-substituted series. **Conclusions**: These findings position *N*,*N′*-disubstituted diamines as a viable scaffold for Chagas disease drug discovery. However, further optimization is required to enhance selectivity, achieve trypanocidal effects, and better understand the underlying mechanisms of action.

## 1. Introduction

Neglected Tropical Diseases (NTDs) are a group of diseases that affect over one billion people worldwide, primarily impacting low-income populations in developing countries [1]. These diseases are caused by a wide range of etiological agents, including parasites, bacteria, fungi, and viruses. Among them, Chagas disease, human African trypanosomiasis (HAT), and leishmaniasis, caused by trypanosomatid parasites, are of particular concern and have been identified by the World Health Organization (WHO) as targets for elimination as public health problems [1].

Chagas disease, caused by *Trypanosoma cruzi*, is endemic to the Americas and poses a major public health challenge in the region. It is estimated that more than 7 million people are currently infected, with approximately 10,000 deaths each year [2]. The primary mode of transmission is vector-borne, with *Triatoma infestans* being the most significant vector. However, due to the success of vector control campaigns, other transmission routes including blood transfusion, congenital, and food contamination have become more prominent [3,4]. These alternative routes explain the presence of Chagas disease in non-endemic countries without vector populations such us Canada, United States, Spain and Australia, among others [4].

Clinically, Chagas disease presents two distinct stages: an acute stage characterized by high parasite load, and a chronic stage with low parasitemia [5]. There are only two available treatments, benznidazole and nifurtimox, both used as monotherapy. While these drugs are effective in treating the acute phase, their efficacy is significantly reduced in the chronic stage.

*T. cruzi*, like other trypanosomatid parasites, undergoes different stages in its life cycle, which are clearly distinguished by cell morphology, being of importance the position, length and attachment point of the flagellum, as well as the relative locations of the nucleus and kinetoplast [6]. The most relevant stages of the life cycle are the extracellular epimastigote and trypomastigote stages, and the intracellular amastigote stage. Since the intracellular amastigote stage is the main form responsible for tissue infection and disease pathology, drug candidates should demonstrate activity against this stage. However, many drug development programs begin screening against the extracellular stages due to their accessibility and lower infection risk. This complexity is further complicated by the significant genetic variability of *T. cruzi*, which is grouped into six discrete typing units (DTUs) [7].

Given the critical need for new drug treatments for Chagas disease as well as for leishmaniasis, anti-trypanosomatid drug discovery programs have been increasingly driven by organizations such as the Drug Neglected Disease Initiative (DNDi), [8,9] the Tres-Cantos Open Lab Foundation (TCOLF) [10] and the Wellcome Centre for Anti-Infectives Research (WCAIR) [11], among others. Still, Chagas disease portfolio is very limited, and no significant progress has been made, with non-new chemical entities being evaluated in Phase II clinical trials [12].

Polyamines are essential compounds for the survival of *T. cruzi* because they are involved in relevant processes, such us protein synthesis, stress resistance, infection establishment and redox balance, among others. The parasite is not able to synthesize polyamines de novo, because it lacks the enzymes arginine decarboxylase and ornithine decarboxylase [13]. Therefore, *T. cruzi* can only obtain these compounds from the extracellular environment via transport processes, being critical for survival of the parasite. TcPAT12, also known as TcPOT1, is the only functionally validated polyamine transporter identified in *T. cruzi* to date, belonging to the TcAAAP (*Trypanosoma cruzi* Amino Acid/Auxin Permeases) family, which is absent in mammals [14,15].

**Figure 1:**
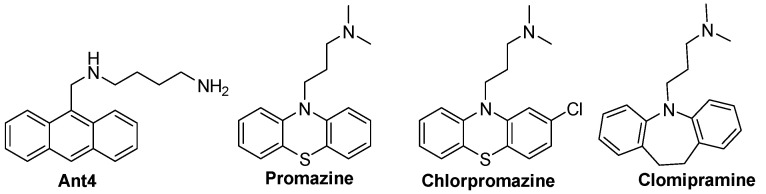
Structures of polyamine transport inhibitors in *T. cruzi*.

Several polyamine transport inhibitors have been identified, e.g., **Ant4**, an anthracene-putrescine conjugate with potent trypanocidal effect on trypomastigotes, the bloodstream stage of *T. cruzi*, with an IC_50_ in the nanomolar range. Furthermore, using **Ant4** as a reference molecule in a ligand-based virtual screening, were identified three inhibitors of polyamine transport: promazine, chlorpromazine, and clomipramine (Figure 1) [16]. All these compounds are approved for use in humans and presented activities against *T. cruzi* parasites similar to **Ant4**.

Previously, our group reported a library of 30 *N*,*N′*-disubstituted diamines, with several compounds displaying activity against trypanosomatids (*T. cruzi*, *L. donovani* and *T. brucei*) and apicomplexans (*P. falciparum* and *T. gondii*) [17]. Since we identified hits by phenotypic biological assay, the mechanisms of action of these compounds in the various agents remain unknown.

Despite the urgent need for new antichagasic agents with improved efficacy and novel mechanisms of action, the therapeutic options for Chagas disease remain limited. In this context, polyamine metabolism and transport have emerged as attractive targets in *Trypanosoma cruzi*. Building on our previous work on a library of polyamine-based diamines, the present study aims to advance this library within the antichagasic drug discovery pipeline. To this end, we evaluated the library’s activity against the intracellular amastigote stage and assessed trypanocidal activity, both critical for therapeutic efficacy. Moreover, to explore potential mechanisms of action, selected compounds were tested as inhibitors of polyamine transport, given the polyamine scaffold of these diamines. We also employed a complementary approach to study compound internalization using a fluorescent analog.

## 2. Results and Discussion

**Scheme 1:**
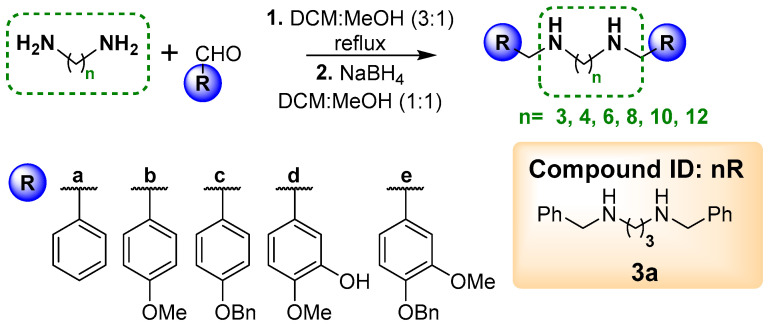
Synthesis of *N*,*N′*-disubstituted diamines library.

As described above, we previously reported the synthesis of 30 *N*,*N′*-disubstituted diamines by reductive amination using diverse 1,ω-aliphatic diamines with carbon chain lengths varying from 3 to 12 (3, 4, 6, 8, 10, and 12) with substituted benzaldehydes (Scheme 1). The aldehydes included benzaldehyde and its oxygenated derivatives: 4-methoxy-, 3-hydroxy-4-methoxy-, 4-benzyloxy-, and 3-methoxy-4-benzyloxy-benzaldehyde. Compound IDs are assigned first by the number of carbons in the diamine chain, then by the code of the respective aldehyde.

The diamine library displayed several promising compounds with nanomolar activity against *T. cruzi* CL-Brener (epimastigote, DTU TcVI) with selectivity index above 10 (Table 1) [17]. Compounds **12a**, **12d** and **12e**, which had not previously been tested against *T. cruzi* epimastigotes, were evaluated and exhibited pIC_50_ of 6.25, 5.40 and 6.01, respectively. Therefore, to advance hit validation, it was necessary and critical to test the library’s activity on *T. cruzi* amastigotes, the clinically relevant intracellular stage.

**Figure 2:**
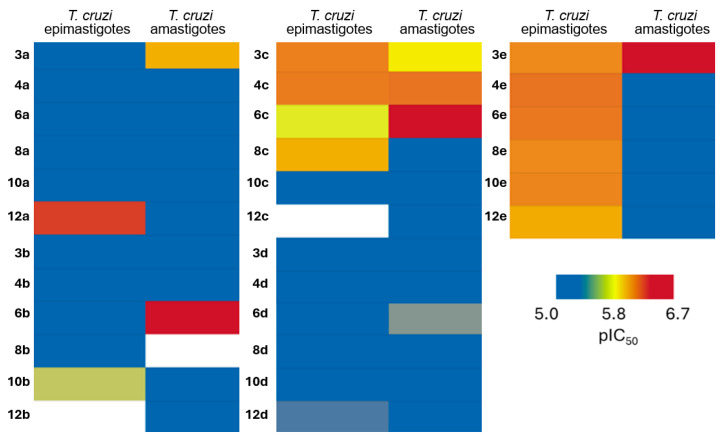
*T. cruzi* epimastigotes and amastigotes activity heatmap. Compounds **8a**, **8c**, **10c**, **10d** exhibited pIC_50_ values of 4.85, 4.92, 4.85 and 4.89, respectively, against amastigote *T. cruzi*.

The inhibition results for *T. cruzi* CL-Brener amastigotes are exhibited in Table 1. Eleven derivatives (37% of the library) displayed pIC_50_ above 4.70 (maximum concentration tested of 20 μM). Among these, five compounds displaying pIC_50_ ≥ 6.0 (**3a**, **3e**, **4c**, **6b** and **6c** with values of 6.00, 6.74, 6.14, 6.70 and 6.44, respectively) and selective index (SI) above 10 (except for **4c**, SI = 8.10). To compare *T. cruzi* biological activity against epimastigote and amastigote stages, we prepared a heatmap of activities (Figure 2), which showed no clear correlation between potencies against epimastigotes and amastigotes. For example, compounds **3a**, **6b** and **6d**, which were inactive on epimastigotes, showed potent activity in the amastigote stage, with pIC_50_ of 6.0, 6.70 and 5.49. In contrast, several compounds what were active on *T. cruzi* epimastigotes were inactive in amastigotes (e.g., **12a**, **10b**, **6e**, **8e** and **10e**). Some derivatives were active against both stages, such as diamines **3c**, **4c**, **6c** and **3e**. These results are consistent with documented evidence that activity in the epimastigote stage is not usually the appropriate filter for identifying hits in Chagas drug discovery. However, epimastigote assays remain useful as a first filter. For example, polyamine inhibitors mentioned above, promazine, chloropromazine and clomipramine displayed only moderate activity against epimastigote (pIC_50_, 4.46, 4.38 and 4.40) but potent for amastigote (pIC_50_, 5.42, 5.72 and 5.54) [16]. Similarly, benznidazole (the standard drug for Chagas disease) presents moderate activity on epimastigotes (pIC_50_= 4.56) but is much more effective against amastigotes (pIC_50_ = 6.71) (Table 1).

A detailed structure–activity relationship (SAR) analysis revealed clear trends associated with both diamine chain length and aromatic substitution. Compounds bearing short to medium diamine chains (3, 4 and 6 carbon atoms) were the most potent against intracellular amastigotes (**3a**, **3c**, **3e**, **4c**, **6b** and **6c**), indicating that shorter diamine chains favor amastigote inhibition.

Within the series containing small aromatic substituents, namely the unsubstituted (a) and 4-methoxy (b) series, biological activity was strongly dependent on the diamine chain length. In the unsubstituted series (a), compound **3a** showed the highest potency (pIC_50_ = 6.00), whereas all other analogues displayed pIC_50_ values below 5.00. In the 4-methoxy series (b), the optimal diamine chain length corresponded to six carbons, as compound **6b** exhibited a pIC_50_ of 6.70, while the remaining analogues were inactive. In contrast, analogues from series a and b with longer diamine chains (**12a** and **10b**) were more active against epimastigotes.

In the 3-hydroxy-4-methoxy series (d), none of the compounds showed pIC_50_ values above 6.00 against either parasite stage, identifying this subset as the least promising within the library. For the 4-benzyloxy series (c), four out of six derivatives were active against both stages of *T. cruzi*. Notably, compound **6c** was the most active derivative against amastigotes, whereas compounds **3c**, **4c** and **8c** exhibited comparable activity against epimastigotes.

Compounds from the 4-benzyloxy-3-methoxy series (e) displayed similar activity against epimastigotes; however, only derivatives bearing shorter diamine chains (**3e** and **4e**) retained appreciable activity against amastigotes.

Taken together, these results indicate that shorter diamine chains generally enhance activity against *T. cruzi* amastigotes, while the combined effect of diamine chain length and aromatic substitution pattern plays a key role in modulating stage-specific potency.

**Figure 3:**
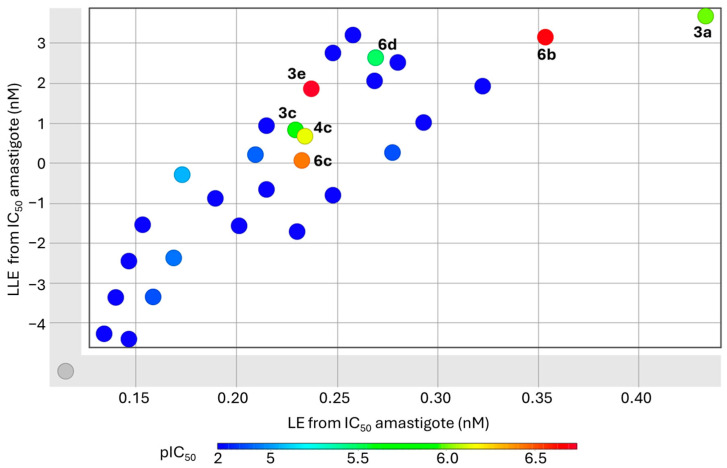
Comparison of compound’s LE, LLE and pIC_50_ values against *T. cruzi* amastigotes.

To evaluate the efficiency of the hits, we assessed ligand efficiency (LE) and lipophilic ligand efficiency (LLE) for the library. Figure 3 presents the LE, LLE, and pIC_50_ values for each compound. Among them, analogs **3a** and **6b** had the best balance, with LE > 3.0 and LLE > 0.03 values exceeding 3.0 and 0.3, respectively, and pIC_50_s > 6.0 against amastigotes. Both **3a** and **6b** were also the most potent in their respective aldehyde series (benzaldehyde and 4-OMe-benzaldehyde). In contrast, the 4-benzyloxy-benzyl series contained more active compounds; however, due to its higher molecular weight and lipophilicity, it generally showed lower LE and LLE values compared to **3a** and **6b**.

Next, we selected nine analogs for further studies: **3a**, **12a**, **6b**, **3c**, **4c**, **6c**, **8c**, **3e** and **6e**. Selection criteria included analogs active in both stages (**3c**, **4c**, **6c** and **3e**) and analogs active in only one stage (epimastigote: **12a**, **8c** and **6e**; amastigote: **3a** and **6b**).

**Figure 4:**
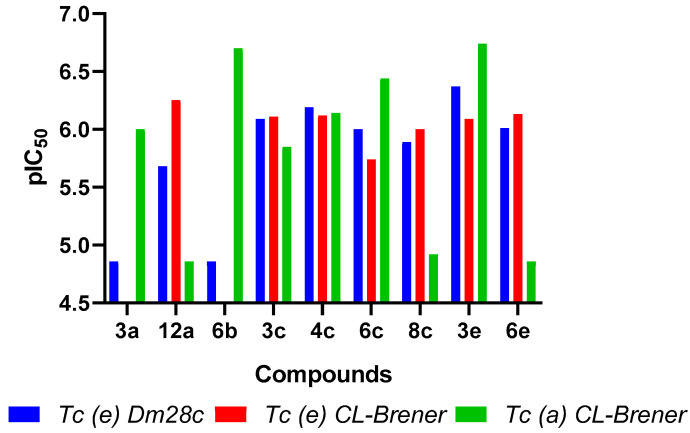
Antichagasic activity of selected hits.

We then tested these analogs against the Dm28c strain (DTU TcI), a genotype relevant in South America. The results (Table 2 and Figure 4) showed similar activity profiles between CL-Brener and Dm28c [18,19]. Overall, comparison of pIC_50_ values between CL-Brener and Dm28c revealed no consistent shift in strain susceptibility. Most analogs showed only minor differences (typically within ~0.1–0.3 log units) that are unlikely to be biologically meaningful. The largest divergence was observed for **12a** (pIC_50_ 5.68 in CL-Brener vs. 6.25 in Dm28c; Δ = +0.57), whereas **3e** showed the greatest shift in the opposite direction (6.37 vs. 6.09; Δ = −0.28). Taken together, these data indicate that the potency of this series is essentially conserved across these two genetically distinct *T. cruzi* strains.

**Figure 5:**
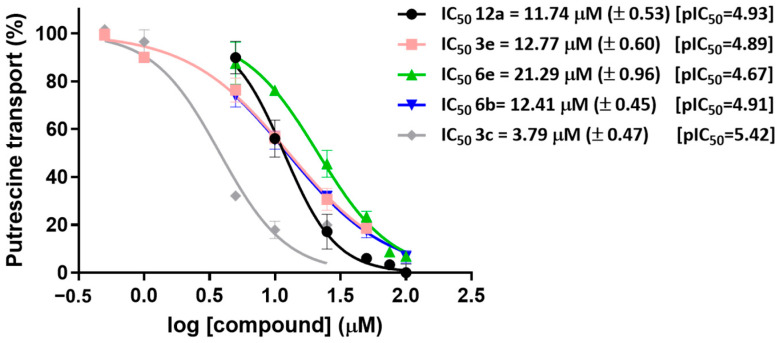
Calculation of *N*,*N′*-disubstituted diamines concentrations required to inhibit 50% of polyamine transport activity (IC_50_). Putrescine transport was measured using epimastigote cultures (10^7^ parasites) and 5 μM [^3^H]-putrescine, in the presence of different concentrations of the compounds between 0 and 100 µM. The IC_50_ values were calculated by non-linear regression using the GraphPad Prism software.

To investigate the action mechanism, we evaluated the ability of nine diamine analogs to inhibit putrescine transport in *T. cruzi* CL-Brener epimastigotes. All analogs inhibited putrescine uptake at 25 μM (Table 3), but IC_50_ values could only be determined for five compounds (Figure 5): **12a**, **6b**, **3c**, **3e** and **6e** that exhibited pIC_50_s of 4.93, 4.91, 5.42, 4.89, and 4.67, respectively.

A comparison between epimastigote growth inhibition and putrescine transport inhibition suggests that poly-amine transport may not be the primary target for all compounds. For instance, although compounds **12a** and **6b** both inhibited putrescine transport by nearly 70% at 25 μM, their effects on parasite growth differed substantially. Nonetheless, transport inhibition seems to play a more direct role in the activity of compound **3c**, which showed the highest potency (pIC_50_ = 5.42). Compounds **12a**, **6b**, **3e**, and **6e** displayed moderate inhibition of transport and, if a polypharmacological mode of action is involved, such effects may still contribute significantly to their overall antiparasitic activity.

Taken together, these observations indicate that inhibition of polyamine transport alone is insufficient to explain antiparasitic activity across the entire compound series and should be considered as one of several contributing mechanisms rather than a sole determinant of efficacy.

Next, we aimed to determine whether the library’s activity is trypanocidal, a key requirement for in vivo efficacy. To this end, we employed the MTS assay (Medium Throughput Screening assay developed by Doyle et al. [20], which measures the IC_50_ against *T. cruzi* Y strain in infected muscle cells, while also providing information on compound cytotoxicity within the same culture system.

The MTS assay involves prolonged exposure of intracellular *T. cruzi* amastigotes, cultured in BESM cells, to the test compound. The duration of the culture (in days) is recorded from the day of compound addition until the emergence of trypomastigotes in the supernatant. Controls include benznidazole (10 μM), posaconazole (12 μM), and K777 (10 μM). Treatments with diamine compounds were carried out at concentrations of 10, 5 and 2.5 μM.

The assay is monitored over a 40-day period, with medium changes every two days. From day 15 onward, the RPMI medium supplemented with the compound is no longer replaced, and only the RPMI medium is renewed until completing 40 days, with the appearance of trypomastigotes and the viability of the culture being monitored every two days. Compounds with low activity typically allow trypomastigote release before day 6. In contrast, trypanostatic compounds delay parasite emergence until between days 6 and 20. Compounds classified as trypanocidal, such as posaconazole and K777, prevent trypomastigote appearance throughout the entire 40-day observation period despite continued host cell growth.

Due to its extended duration and resource requirements, the MTS assay is not suitable for high-throughput screening. Instead, it is employed to assess compound mode of action and to serve as a secondary filter prior to in vivo evaluation.

Unfortunately, the results indicated that none of the selected compounds demonstrated trypanocidal activity in the MTS assay. Surviving days are exhibited in Table 4 at a diamine concentration of 2.5 μM. Increased diamine treatment concentrations (5 or 10 μM) displayed equal or less days of trypomastigote appearance. The 4-benzyloxy and 3-methoxy-4-benzyloxy derivatives tested (**3c**, **4c**, **10c**, **6e**, and **8e**) exhibited cytotoxicity, except for **4e**, which showed a trypanostatic profile. These findings were consistent with the Vero cell assays, where compounds **3c**, **4c**, and **8e** also displayed cytotoxic effects. It is likely that compounds **10c** and **6e** are cytotoxic in Vero cells as well, but only at concentrations higher than those tested.

Compound **3a**, despite showing potent in vitro activity, displayed a trypanostatic rather than trypanocidal effect in the MTS assay, suggesting it would be insufficient to achieve parasite clearance in vivo. Interestingly, even compounds that were inactive at the highest concentration tested (20 μM) exhibited a trypanostatic effect similar to that of **3a**. Although none of the compounds achieved complete parasite clearance in the MTS assay, this outcome does not necessarily preclude in vivo efficacy. The MTS assay represents a highly stringent in vitro model that requires prolonged exposure under fixed conditions and does not account for pharmacokinetic behavior, tissue distribution, metabolic activation, or host immune responses, all of which can contribute to parasite control in vivo. Indeed, several antitrypanosomal agents display trypanostatic or partial activity in vitro while showing efficacy in animal models or clinical settings when adequate exposure is achieved.

In this context, the observed trypanostatic profiles, particularly for compound **3a**, suggest that sustained drug exposure or combination strategies could be required to achieve parasite clearance. Further evaluation using alternative host cells, extended dosing regimens, or in vivo models will be necessary to fully assess the therapeutic potential of these compounds.

As a complementary approach, we evaluated compound internalization in *T. cruzi* epimastigotes using a fluorescent analog. This strategy provides insights into the compound’s mechanism of action, particularly regarding cellular uptake and potential target localization. However, the use of fluorescent tags often requires structural modifications that may alter the steric and electronic properties of the parent compound, potentially affecting its biological activity.

Among the available fluorescent probes, we selected 7-nitrobenzofurazan (NBD) due to its small size, low polarity, cost-effectiveness, and ease of chemical incorporation [21].

**Scheme 2:**
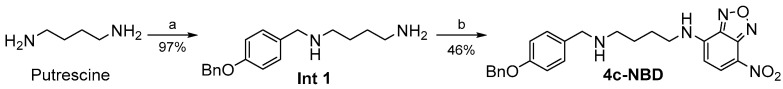
Synthesis of NBD labeled analog. **Conditions:** (a) 4-(benzyloxy)benzaldehyde, DCM/MeOH (3:1) reflux, 18h; then NaBH_4_, DCM/MeOH (1:1), 0 °C 2h; (b) 1-NBD-Cl, NaHCO_3_; AcCN, 0 ° to 60 °C 2h then 60 °C, 5h.

The synthesis of the fluorescent analog (**4c-NBD**) is shown in Scheme 2 and the mono reductive amination with 4-benzyloxybenzaldehyde of putrescine and the aromatic nucleophilic substitution with 4-chloro-7-nitrobenzofurazan to afford the desired fluorescent product.

**Figure 6:**
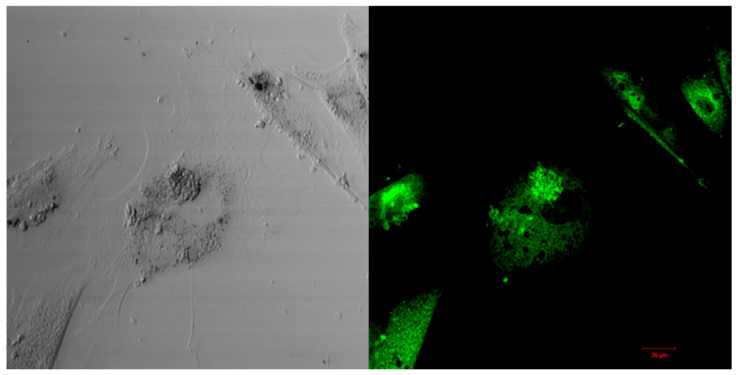
Confocal microscopy images of bovine embryo skeletal muscle (BESM) infected with *Trypanosoma cruzi* amastigotes and treated with **4c-NBD**. The left panel shows the corresponding differential interference contrast (DIC) image, and the right panel shows the fluorescence channel, where intracellular accumulation of **4c-NBD** (green) can be observed within the amastigotes.

Activity against *T. cruzi* epimastigotes was evaluated; however, the compound did not exhibit antiparasitic activity at the maximum concentration tested of 50µM. This loss of activity may be attributed to the reduced basicity of the nitrogen atom adjacent to the NBD group. We hypothesize that the mechanism requires binding through two basic nitrogens acting as anchoring points on the molecular target, which is not possible in this case, thus explaining the lack of activity. Alternatively, differences in electron distribution between the NBD moiety and the original 4-benzyloxybenzaldehyde fragment may also contribute to the lack of efficacy. Despite this, fluorescent microscopy assays yielded satisfactory results. Confocal imaging revealed accumulation of **4c-NBD** in intracellular amastigotes (Figure 6, right panel). The precise site of accumulation within the parasite remains unclear, but it may be relevant to the compound’s mode of action. Notably, the lack of fluorescence accumulation at the plasma membrane argues against a mechanism based on transporter blockade. Instead, the compound appears to cross cellular membranes and act at intracellular sites, supporting the hypothesis of a non-membrane-localized target. Nevertheless, is not discarded activity in both intracellular targets and inhibition of polyamine transport, similarly to Ant4 and chlorpromazine [16]. In line with this, a related study employing Spermine-NBD as a fluorescent probe demonstrated that the compound is internalized by *Leishmania donovani* promastigotes and accumulates inside the parasites rather than remaining at the plasma membrane [22]. These observations confirm that the aliphatic diamine scaffold facilitates selective delivery into the parasite. Hence, these findings provide experimental evidence that the series of dibenzyl-aliphatic diamines can effectively reach the intracellular stage of the parasite, which is essential for their antiparasitic activity.

Since it is possible that the diamine scaffold acts as a polyamine transport inhibitor and can also be internalized to interact with intracellular targets, a polypharmacological mechanism of action might be involved. This hypothesis is further supported by the significant differences observed between transport inhibition and growth inhibition assays.

Several polyamines and amine derivatives have been reported to display mechanisms of action unrelated to polyamine biosynthesis. Due to their polycationic nature, these compounds tend to interact with electron-rich intracellular components such as mitochondria and DNA targets. For example, Martín-Escolano et al. described an aryl polyamine derivative that induced a mitochondria-dependent bioenergetic collapse and oxidative stress through inhibition of the Fe-SOD enzyme [23]. Likewise, the antitubercular diamine compound SQ109, which also exhibits potent antichagasic activity, has been shown to act through a polypharmacological mechanism involving both the collapse of the mitochondrial membrane potential and inhibition of squalene synthase [24].

In addition, other cationic moieties, such as phosphonium salts, have demonstrated the ability to interact with electron-rich targets. For instance, Manzano et al. reported a 4-hydroxyphenyl phosphonium salt derivative with potent in vivo activity in a mouse model of visceral leishmaniasis, acting through a mitochondrial mechanism that includes disruption of energy metabolism and increased reactive oxygen species (ROS) production [25].

Based on these findings, our next step in the mechanism of action studies will focus on investigating whether our diamine derivatives interact with mitochondrial-related targets.

Overall, the present study was conceived as an exploratory effort to identify structure–activity relationships and obtain preliminary biological insights into *N*,*N′*-disubstituted diamines active against *T. cruzi*. Although the results reveal relevant antiparasitic, transport-related, and cytotoxicity trends, the experimental assays were not designed to conclusively define the molecular target(s) or mechanism(s) of action. Moreover, the absence of full trypanocidal activity in short-term assays and the limited number of host cell models evaluated constrain direct extrapolation to in vivo efficacy. Future work involving extended exposure times, additional cellular models, and dedicated mechanistic studies will be required to further clarify these aspects.

## 3. Materials and Methods

### 3.1. Biological Assays

#### 3.1.1. Host Cell Culture and Infection with *T. cruzi* Cl Brener

For intracellular amastigote assays, cardiac myocytes were cultured in RPMI-1640 medium supplemented with 5% heat-inactivated fetal bovine serum (FBS Internegocios, Mercedes, Buenos Aires, Argentina). Cardiac myocytes were seeded at a density of 1 × 10^5^ cells/cm^2^ and incubated overnight in 384-well assay plates. Plates were sealed and incubated at 37 °C in a 5% CO_2_ atmosphere.

Trypomastigotes of *T. cruzi* (Cl Brener strain) were maintained by weekly passages in primary cultures of bovine embryo skeletal muscle cells (BESM cells). Cultures were kept at 37 °C in RPMI-1640 supplemented with 5% heat-inactivated horse serum and 5% CO_2_. The infection cycle of this strain under these conditions is 6–7 days. Once released, trypomastigotes were harvested from the supernatant and used for cardiac myocytes infection.

Cells were infected at a 1:1 parasite-to-host cell ratio (not exceeding 1 × 10^5^ trypomastigotes/mL). After infection, plates were sealed and incubated at 37 °C in 5% CO_2_.

#### 3.1.2. Antichagasic Activity Against *T. cruzi* Cl Brener Amastigotes

Stock solutions (10 mM) of diamine compounds were prepared in DMSO (Sigma-Aldrich, St. Louis, MO, USA) and diluted to 10 μM in RPMI-1640 + 5% FBS before use (working solution). Stocks were stored at –20 °C and working dilutions at 4 °C throughout the experiments. Each assay included the following controls: posaconazole (100 nM), benznidazole (1 mM), no-treatment (negative control), and uninfected cells (background control), in triplicate per treatment.

Compounds were added to each well either at a fixed concentration or serially diluted for pIC_50_ determination. Infected cultures were incubated with compounds for 48 h at 37 °C. Afterwards, the inhibitor was removed by washing the cells at least three times with inhibitor-free medium and the medium was replaced.

Following incubation, cells were washed with PBS, fixed with 4% formaldehyde for 30 min, and stained with 300 nM 4′,6-diamidino-2-phenylindole (DAPI, Sigma-Aldrich, St. Louis, MO, USA) for 2 h. Excess stain was removed by washing with PBS.

Images were acquired using an GE Healthcare Life Sciences INCell Analyzer automated epifluorescence microscope (Marlborough, MA, USA) equipped with excitation/emission filters of 350 ± 50 nm and 460 ± 40 nm, respectively. A 20× objective was used for image capture. Image analysis was performed using Developer Toolbox 1.7 software, which was programmed to identify host cell nuclei (area > 250 µm^2^) and exclude kinetoplastid nuclei (fluorescent area ~1 µm^2^). Parasite quantification was performed within a defined area of 700 to 2000 µm^2^, corresponding to the cellular boundary. Extracellular parasites and false staining artifacts (false positives) were excluded. The percentage of growth inhibition was calculated using the following formula:

[1 − (P/hc_x_ − P/hc^+^)/(P/hc_2_ − P/hc^+^)] × 100

P/hc_x_ = parasite count per host cell in treated wells

P/hc^+^ = parasite count per host cell in positive control wells (benznidazole, posaconazole)

P/hc_2_ = parasite count per host cell in negative control wells

#### 3.1.3. Parasite Culture, Growth and Antichagasic Activity in *T. cruzi* Epimastigotes

Dm28c strain epimastigotes were routinely maintained at logarithmic phase of growth in Liver Infusion Tryptose medium (LIT: 5 g⋅L^−1^ liver infusion broth, 5 g·L^−1^ bacto-tryptose, 68 mM NaCl, 5.3 mM KCl, 22 mM Na_2_HPO_4_, 0.8% (*w*/*v*) glucose) supplemented with 10% Fetal Bovine Serum and 5 µM hemin at 28 °C.

CL Brener epimastigotes were grown at 28 °C in plastic flasks (25 cm^2^), containing 5 mL of brain-heart infu-sion-tryptose (BHT) medium supplemented with 10% fetal calf serum (FCS), 100 U.mL^−1^ penicillin, 100 μg.mL^−1^ streptomycin and 20 μg.mL^−1^ hemin [26].

Parasites were incubated with serial dilutions of test compounds to determine IC_50_s activities. One hundred micro-liters of culture (2 × 10^6^ epimastigotes/mL) were incubated during 72 h at 28 °C in the presence of different concentrations of the diamines in triplicate. After incubation, cultures were observed in an inverted microscope Olympus CK2 (Tokyo, Japan) and parasite concentrations were measured with an automatic hematology analyzer (Counter 19; Wiener Lab, Rosario, Argentina) via WBC channel reads. pIC_50_s were determined using GraphPad Prism 5 software, plotting the Log [compound] vs. [epimastigotes] and applying a non-linear regression (log(inhibitor) vs. response—Variable slope).

#### 3.1.4. Polyamine Transport Assays in *T. cruzi* CL-Brener Epimastigotes

Aliquots of CL-Brener epimastigote cultures (10^7^ parasites) were centrifuged at 8000× *g* for 30 s and washed once with phosphate-buffered saline (PBS). Cells were resuspended in 0.1 mL PBS and the assay started by the addition of 0.1 mL of the transport mixture containing [^3^H]-putrescine (PerkinElmer’s NEN Radiochemicals; 0.4 μCi) in the presence of 5 µM putrescine and different concentrations of the indicated compounds. Following incubation during 10 min at 28 °C, the transport reaction was stopped by adding 1 mL of ice-cold PBS. Cells were centrifuged as indicated above and washed twice with ice-cold PBS. Cell pellets were resuspended in 0.2 mL of water and counted for radioactivity in UltimaGold XR liquid scintillation cocktail (Packard Instrument Co., Meridien, CT, USA) [27]. Non-specific uptake and carry over were assayed without incubation (T0) or incubated at 4 °C. Cell viability was assessed by direct microscopic examination.

All the experiments were done at least in triplicates and results presented here are representative of three independent assays. pIC50 values were obtained by non-linear regression of dose response logistic functions, using GraphPad Prism 6.01.

#### 3.1.5. Medium Throughput Screening Assay

Bovine embryo skeletal muscle (BESM) cells were cultured in RPMI-1640 medium supplemented with 5% heat-inactivated fetal calf serum (FCS). To arrest cell proliferation, macrophages were irradiated 15 min at approximately 1000 rad. After irradiation, macrophages (1 × 10^5^ cells/cm^2^) were seeded in 12-, 24-, or 48-well tissue culture plates and incubated overnight at 37 °C in a humidified atmosphere with 5% CO_2_.

Diamine compounds were prepared as 10 mM stock solutions in DMSO and diluted in RPMI + 5% FBS to a working concentration of 10, 5 and 2.5 µM. Assays included the following controls: posaconazole-treated (10 μM), K777-treated (12 μM), benznidazole-treated (10 μM), infected but untreated, and non-infected macrophages (n = 3 per condition).

Trypomastigotes of *T. cruzi* (Y strain) were maintained by weekly passages in primary cultures of BESM cells. Cultures were incubated at 37 °C in RPMI-1640 medium supplemented with 5% heat-inactivated horse serum and 5% CO_2_. The parasite life cycle under these conditions is 6–7 days. Once released into the supernatant, trypomastigotes were collected and used for infection of host cells.

Parasites were added to the cultures at a 1:1 parasite-to-host cell ratio, not exceeding 1 × 10^5^ trypomastigotes/mL. After 2 h of incubation at 37 °C, cells were washed at least three times with inhibitor-free medium and, subsequently, RPMI-1640 containing the test compound (10 µM) was added to each well. Assays included the following controls: posaconazole-treated (10 μM), K777-treated (12 μM), benznidazole-treated (10 μM), infected but untreated, and non-infected macrophages (n = 3 per condition).

The culture medium, containing either diamines derivatives or controls, was replaced every 48 h for a period of 12 days. After this period, cells were maintained in RPMI-1640 without inhibitor, with medium changes every 48 h until day 27 post-infection.

Cultures were monitored every 48 h using phase-contrast microscopy. The presence of trypomastigotes in the culture supernatant was interpreted as completion of the *T. cruzi* intracellular cycle and was used as the exclusion criterion for wells in which infection persisted. The duration of the experiment was up to 40 days, or until trypomastigotes were released from BESM cells, whichever occurred first.

Efficacy evaluation criteria:

Low efficacy: Compounds with no activity allowed the appearance of free trypomastigotes in the supernatant by day 6 post-infection, similarly to untreated controls.

Trypanostatic effect: Compounds that delayed but did not prevent parasite development (>6 days to detect extracellular trypomastigotes) were considered trypanostatic.

Trypanocidal activity: Compounds that completely cleared the infection showed no intracellular or extracellular parasites by day 40 post-infection, comparable to control drugs (posaconazole, benznidazole and K777).

#### 3.1.6. Confocal Microscopy

Alive confocal images were acquired with a Zeiss confocal micro-scope (Axiovert 200M, LSM 510 Meta; Carl Zeiss Inc., Thornwood, NY, USA) using a 40×, 1.4 numerical aperture oil objective. Images were collected at 1024 × 1024 frame resolution with a pinhole of 0.75 Airy units.

### 3.2. Synthesis

#### 3.2.1. General Information

^1^H and ^13^C NMR spectra were acquired on a Bruker Avance II 300 MHz (75.13 MHz) (Bruker BioSpin, Billerica, MA, USA) using CDCl_3_ as solvent. Chemical shifts (δ) were reported in ppm downfield from tetramethylsilane (TMS) at 0 ppm as internal standard and coupling constants (*J*) are in hertz (Hz). Chemical shifts for carbon nuclear magnetic resonance (^13^C NMR) spectra are reported in parts per million relatives to the center line of the CDCl_3_ triplet at 76.9 ppm. The following abbreviations are used to indicate NMR signal multiplicities: s = singlet, d = doublet, t = triplet, q = quartet, p = pentet, h = hextet, m = multiplet, br = broad signal. High-resolution mass spectra (HRMS) were recorded on a Waters Synapt XS (Milford, MA, USA) with lock spray source. Chemical reagents were purchased from commercial suppliers and used without further purification, unless otherwise noted. Solvents were analytical grade or were purified by standard procedures prior to use. Yields were calculated for material judged homogeneous by thin layer chromatography (TLC) and nuclear magnetic resonance (^1^H NMR). All reactions were monitored by thin layer chromatography performed on silica gel 60 F_254_ pre-coated aluminium sheets, visualized by a 254 nM UV lamp, and stained with an ethanolic solution of 4-anisaldehyde (Sigma-Aldrich, St. Louis, MO, USA). Column flash chromatography was performed using silica gel 60 (230–400 mesh). All final compounds were purified to ≥95% purity, as determined ^1^H and ^13^C NMR analysis.

#### 3.2.2. Synthesis of Fluorescence Analog

##### Synthesis of N^1^-(4-(Benzyloxy)benzyl)butane-1,4-diamine (Int 1)

To a solution of 1,4-butylenediamine (350 mg; 3.97 mmol) in anhydrous DCM:MeOH (3:1), 4-(benzyloxy)benzaldehyde (421 mg; 1.99 mmol) was added and the reaction was refluxed for 18 h; The reaction was then cooled in an ice bath to below room temperature, and anhydrous MeOH was added until a 1:1 DCM:MeOH equivalence was reached. Sodium borohydride (90 mg; 2.38 mmol) was added as a reducing agent and left to stir until it reached room temperature for 2 h. The solvent was evaporated under vacuum and the reaction redissolved in DCM. Finally, 1M HCl was added until a pH of 2–3 was reached, left to stir for 30 min, and then NH_4_OH was added until a pH of 10–12 was reached. The organic phase was filtered through celite and washed with distilled water (3 × 15 mL), dried with Na_2_SO_4_, filtered through a DCM:EtOH:TEA (20:79:1) and concentrated with a yield of (266 mg; 0.93mmol) 47% for a yellowish-white solid.

^1^H NMR (CDCl_3_): δ = 7.47–7.27 (m, 5H); 7.23 (d, *J* = 8.6 Hz, 2H); 6.93 (d, *J* = 8.6 Hz, 2H); 5.05 (s, 2H); 3.73 (s, 2H); 2.70 (t, *J* = 6.7 Hz, 2H); 2.64 (t, *J* = 6.7 Hz, 2H); 1.70–1.40 (m, 4H). ^13^C NMR (CDCl_3_): δ = 158.0 (C); 137.0 (C); 131.8 (C); 129.6 (CH); 128.6 (CH); 128.0 (CH); 127.5 (CH); 114.8 (CH); 70.0 (CH_2_); 53.0 (CH_2_); 48.7 (CH_2_); 41.5 (CH_2_); 30.7 (CH_2_); 27.2 (CH_2_). ESI-HRMS Calcd for C_18_H_25_N_2_O (M+H^+^): 285.1967; found 285.1978.

##### Synthesis of *N*-(4-(4-(Benzyloxy)benzylamino)butyl)-7-nitrobenzo[c][1,2,5]oxadiazol-4-amine (**4c-NBD**)

N^1^-(4-(Benzyloxy)benzyl)butane-1,4-diamine (28 mg, 0.098 mmol) and sodium bicarbonate (9.9 mg, 0.118 mmol) were dissolved in anhydrous acetonitrile (3 mL). A solution of 4-chloro-7-nitrobenzofurazan (12 mg, 0.059 mmol) in anhydrous acetonitrile (5 mL) was placed in a dropping funnel and added dropwise over 2 h. The reaction mixture was then stirred at room temperature for an additional 5 h. After completion, the solvent was evaporated under reduced pressure, and the residue was dissolved in dichloromethane. The organic phase was washed with distilled water (3 × 15 mL), dried over anhydrous Na_2_SO_4_, filtered, and concentrated. Purification by column chromatography (DCM/EtOH/TEA, 98:1:1) afforded a reddish-yellow solid in 18% yield.

^1^H NMR (CDCl_3_): δ = 8.45 (d, *J* = 8.6 Hz, 1H, NBD), 7.44–7.25 (m, 5H, BnO-Bn), 7.25 (d, *J* = 8.7 Hz, 2H, BnO-Bn), 6.94 (d, *J* = 8.7 Hz, 2H, BnO-Bn), 6.05 (d, *J* = 8.6 Hz, 1H, NBD); 5.05 (s, 2H, **CH_2_**-O), 3.88 (s, 2H, **CH_2_**-NH), 3.42 (m, 2H, **CH_2_**-NH); 3.27 (m, 2H, **CH_2_**-NH); 1.92 (m, 2H, **CH_2_**-CH_2_); 1.74 (m, 2H, CH_2_-**CH_2_**). ^13^C NMR (CDCl_3_): δ = 158.2 (CH, BnO-Bn), 144.9 (C, NBD), 144.5 (C, NBD), 144.2 (C, NBD), 136.9 (C, BnO-Bn), 136.9 (CH, NBD), 129.8 (CHx2, BnO-Bn); 128.7 (C, BnO-Bn) 128.6 (CHx2, BnO-Bn); 128.0 (C, BnO-Bn); 127.5 (CHx2, BnO-Bn); 124.5 (C, NBD), 115.1 (CHx2, BnO-Bn); 98.2 (CH, NBD); 70.0 (CH2, CH_2_-O), 52.61 (CH_2_, CH_2_-NH), 47.4 (CH_2_), 44.0 (CH_2_), 27.3 (CH_2_), 26.5 (CH_2_). ESI-HRMS Calcd for C_24_H_26_N_5_O_4_ (M+H^+^): 448.1985; found 448.1977.

## 4. Conclusions

This work establishes *N*,*N′*-disubstituted diamines as a promising scaffold for the development of novel antichagasic agents. Several analogs exhibited potent and selective activity against the intracellular amastigote stage of *T. cruzi*, reinforcing the critical importance of stage-specific screening in Chagas disease drug discovery. The clear dissociation between amastigote and epimastigote activity highlights the limitations of relying solely on extracellular models during early-stage compound evaluation.

Mechanistic studies provided further insight into potential modes of action. Compound **3c** significantly inhibited polyamine transport, yet this effect was not observed across all active analogs, suggesting either target promiscuity or alternative pathways of action. To better understand compound uptake and localization, a fluorescent analog was synthesized and assessed by confocal microscopy. The analog demonstrated robust accumulation in intracellular amastigotes, confirming internalization and supporting the feasibility of intracellular targeting. However, its lack of biological activity underscores the importance of preserving specific structure–activity relationships for efficacy.

Importantly, none of the compounds demonstrated trypanocidal activity under prolonged exposure, and several—especially those bearing benzyloxy substituents—exhibited cytotoxicity. Despite these limitations, the scaffold holds clear potential: it is synthetically accessible, biologically validated against the relevant parasite stage, and amenable to rational modification. Future efforts should focus on enhancing selectivity, converting trypanostatic profiles into trypanocidal ones, and advancing lead compounds through scaffold optimization and target deconvolution.

## Figures and Tables

**Table 1:** Amastigote and epimastigote *T. cruzi* activity and cytotoxicity values.

ID	*Tc* (a) ^1^ pIC_50_	*Tc* (e) ^2^ pIC_50_	Ctt ^3^ pIC_50_	SI ^4^ Cit/*Tc* (a)	ID	*Tc* (a) ^1^ pIC_50_	*Tc* (e) ^2^ pIC_50_	Ctt ^3^ pIC_50_	SI ^4^ Cit/*Tc* (a)
**3a**	6.00	<5.00	<4.73	>18.71	**8a**	4.85	<5.00	<4.83	>1.05
**3b**	<4.70	<5.00	<4.82	**-**	**8b**	NT	<5.00	<4.91	-
**3c**	5.85	6.11	5.22	4.29	**8c**	4.92	6.00	<5.05	>0.74
**3d**	<4.70	<5.00	<4.86	**-**	**8d**	<4.70	<5.00	<4.94	-
**3e**	6.74	6.09	5.18	36.94	**8e**	<4.70	6.09	5.34	-
**4a**	<4.70	<5.00	<4.75	**-**	**10a**	<4.70	<5.00	<4.87	-
**4b**	<4.70	<5.00	<4.84	**-**	**10b**	<4.70	5.64	<4.94	
**4c**	6.14	6.12	5.23	8.10	**10c**	4.85	<5.00	<5.07	>0.60
**4d**	<4.70	<5.00	<4.88	**-**	**10d**	4.89	<5.00	<4.97	>0.82
**4e**	5.05	6.14	<5.06	0.30	**10e**	<4.70	6.10	<5.12	
**6a**	<4.70	<5.00	<4.79	**-**	**12a**	<4.70	6.25	<4.90	-
**6b**	6.70	<5.00	5.08	41.60	**12b**	<4.70	NT	<4.97	-
**6c**	6.44	5.74	<5.03	>26.00	**12c**	<4.70	NT	<5.10	-
**6d**	5.49	<5.00	<4.91	>3.83	**12d**	<4.70	5.40	<5.00	-
**6e**	<4.70	6.13	<5.08	**-**	**12e**	<4.70	6.01	<5.14	-
**Benz**	6.71	4.56							

**^1^ *Tc* (a):*** T. cruzi* CL Brener amastigotes, maximum concentration tested = 20 μM. **^2^ *Tc* (e):** *T. cruzi* CL Brener epimastigote, maximum concentration tested = 10 μM. **^3^ Ctt:** Cytotoxicity against Vero cells, maximum concentration tested 4.76 μg/mL. **Benz:** Benznidazole; NT: not tested. ^4^ **SI**: Selectivity index IC_50_ Vero cells/IC_50_ *Tc* (a).

**Table 2:** Antichagasic activity of selected hits.

ID	*Tc* (e) Dm28cpIC_50_	*Tc* (e) CL-Brener ^≠^ pIC_50_	*Tc* (a) CL-Brener pIC_50_	Vero Cells pIC_50_
**3a**	4.86	<5.00	6.00	<4.73
**12a**	5.68	6.25	<4.70	<4.90
**6b**	4.86	<5.00	6.70	5.08
**3c**	6.09	6.11	5.85	5.22
**4c**	6.19	6.12	6.14	5.23
**6c**	6.00	5.74	6.44	<5.03
**8c**	5.89	6.00	4.92	<5.05
**3e**	6.37	6.09	6.74	5.18
**6e**	6.01	6.13	<4.70	<5.08
**Benz**	4.71	4.56	6.71	

(e) epimastigote stage. (a) amastigote stage; ^≠^ maximum concentration tested = 10 μM.

**Table 3:** Effect of *N*,*N′*-substituted diamines on polyamine transport.

ID pIC_50_ *T. cruzi* CL-Brener		Putrescine Transport Inhibition	
		pIC_50_	(%) ± (SD) *
**3a**	<5.00	-	14.28 ± 2.88
**12a**	6.25	4.93	70.04 ± 1.51
**6b**	<5.00	4.91	69.54 ± 6.90
**3c**	6.11	5.42	86.11 ± 6.65
**4c**	6.12	-	65.05 ± 10.89
**6c**	5.74	-	31.45 ± 9.41
**8c**	6.00	-	10.90 ± 2.42
**3e**	6.09	4.89	73.75 ± 4.61
**6e**	6.13	4.67	54.10 ± 6.42

pIC_50_s for the inhibition of putrescine transport and the inhibition (expressed as %) of putrescine transport at 25 µM of each compound in epimastigote stage of *T. cruzi*. * The average of three independent experiments (mean ± standard deviation). * [ID] = 25 µM.

**Table 4:** MTS assayed to assess compounds mode of action.

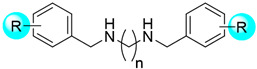					
ID	*n*	R	HTS (pIC50)	MTS *	Result
** 3a **	3	4-H	6.00	20	Trypanostatic
** 4a **	4	4-H	<4.70	20	Trypanostatic
** 10a **	10	4-H	<4.70	20	Trypanostatic
** 3b **	3	4-MeO	<4.70	12	Trypanostatic
** 10b **	10	4-MeO	<4.70	20	Trypanostatic
** 12b **	12	4-MeO	<4.70	20	Trypanostatic
** 3c **	3	4-BnO	5.85	2	Cytotoxic
** 4c **	4	4-BnO	6.14	2	Cytotoxic
** 10c **	10	4-BnO	4.85	2	Cytotoxic
** 4d **	4	4-MeO, 3-OH	<4.70	12	Trypanostatic
** 4e **	4	4-BnO, 3-MeO	5.05	12	Trypanostatic
** 6e **	6	4-BnO, 3-MeO	<4.70	8	Cytotoxic
** 8e **	8	4-BnO, 3-MeO	<4.70	2	Cytotoxic
** Benz ^#1^**				>40	
** Pos ^#2^**				>40	
** K777 ^#3^**				>40	
** Not Treatment **				6	

* Surviving days. Concentration of control drugs used: **Diamine** 2.5 µM; ^#**1**^ **Benz** 10 µM; ^#2^ **Pos** 12 µM ^#3^ **K777** 10 µM.

## Data Availability

The original contributions presented in this study are included in the article/Supplementary Material. Further inquiries can be directed to the corresponding author.

## Supplementary Materials

The following supporting information can be downloaded at: https://www.mdpi.com/article/10.3390/ph19010119/s1, Table S1. Compound’s SMILES. Table S2. Values of compound’s ligand efficiency (LE) and ligand lipophilic efficiency (LLE). Figures S1–S2 and S4–S5 NMR spectra of **INT1** and **4c-NBD**. Figures S3 and S6 ESI-HRMS of **INT1** and **4c-NBD**.

## Abbreviations

The following abbreviations are used in this manuscript:

NTDs I	Neglected Tropical Diseases
HAT	human African trypanosomiasis
WHO	World Health Organization
DNDi	Drug Neglected Disease Initiative
TCOLF	Tres-Cantos Open Lab Foundation
WCAIR	Wellcome Centre for Anti-Infectives Research
DTUs	discrete typing units

## References

[1] WHO (2021). Ending the Neglect to Attain the SDGs: A Roadmap for NTDs.

[2] WHO https://www.who.int/news-room/fact-sheets/detail/chagas-disease-(american-trypanosomiasis).

[3] Antinori S., Galimberti L., Bianco R., Grande R., Galli M., Corbellino M. (2017). Chagas Disease in Europe: A Review for the Internist in the Globalized World. Eur. J. Intern. Med..

[4] Martín-Escolano J., Medina-Carmona E., Martín-Escolano R. (2020). Chagas Disease: Current View of an Ancient and Global Chemotherapy Challenge. ACS Infect. Dis..

[5] Teixeira A.R.L., Nascimento R.J., Sturm N.R. (2006). Evolution and Pathology in Chagas Disease: A Review. Mem. Inst. Oswaldo Cruz.

[6] Kaufer A., Ellis J., Stark D., Barratt J. (2017). The Evolution of Trypanosomatid Taxonomy. Parasit. Vectors.

[7] Higuera S.L., Guhl F., David Ramírez J. (2013). Identification of Trypanosoma Cruzi Discrete Typing Units (DTUs) through the Implementation of a High-Resolution Melting (HRM) Genotyping Assay. Parasit. Vectors.

[8] DNDi. https://Dndi.Org/Research-Development/Portfolio/Open-Chagas/.

[9] DNDi. https://Dndi.Org/News/2013/First-Early-Stage-Research-Latin-America/.

[10] Calderón F., Ballell L., Barros D., Bilbe G., Cammack N., Gabarró R., Halsey A., Heaton P.M., Kang G., Mizrahi V. (2021). Tres Cantos Open Lab: Celebrating a Decade of Innovation in Collaboration to Combat Endemic Infectious Diseases. Nat. Rev. Drug. Discov..

[11] McGonagle K., Tarver G.J., Cantizani J., Cotillo I., Dodd P.G., Ferguson L., Gilbert I.H., Marco M., Miles T., Naylor C. (2022). Identification and Development of a Series of Disubstituted Piperazines for the Treatment of Chagas Disease. Eur. J. Med. Chem..

[12] de Rycker M., Wyllie S., Horn D., Read K.D., Gilbert I.H. (2023). Anti-Trypanosomatid Drug Discovery: Progress and Challenges. Nat. Rev. Microbiol..

[13] Birkholtz L.M., Williams M., Niemand J., Louw A.I., Persson L., Heby O. (2011). Polyamine Homoeostasis as a Drug Target in Pathogenic Protozoa: Peculiarities and Possibilities. Biochem. J..

[14] Carrillo C., Canepa G.E., Algranati I.D., Pereira C.A. (2006). Molecular and Functional Characterization of a Spermidine Transporter (TcPAT12) from Trypanosoma Cruzi. Biochem. Biophys. Res. Commun..

[15] Hasne M.-P., Coppens I., Soysa R., Ullman B. (2010). A High-Affinity Putrescine-Cadaverine Transporter from Trypanosoma Cruzi. Mol. Microbiol..

[16] Reigada C., Sayé M., Phanstiel O., Valera-Vera E., Miranda M.R., Pereira C.A. (2019). Identification of Trypanosoma Cruzi Polyamine Transport Inhibitors by Computational Drug Repurposing. Front. Med..

[17] Panozzo-Zénere E.A., Porta E.O.J., Arrizabalaga G., Fargnoli L., Khan S.I., Tekwani B.L., Labadie G.R. (2018). A Minimalistic Approach to Develop New Anti-Apicomplexa Polyamines Analogs. Eur. J. Med. Chem..

[18] Frédérique B.S., Waleckx E., Barnabé C. (2016). Over Six Thousand Trypanosoma Cruzi Strains Classified into Discrete Typing Units (DTUs): Attempt at an Inventory. PLoS Negl. Trop. Dis..

[19] Velásquez-Ortiz N., Herrera G., Hernández C., Muñoz M., Ramírez J.D. (2022). Discrete Typing Units of Trypanosoma Cruzi: Geographical and Biological Distribution in the Americas. Sci. Data.

[20] Doyle P.S., Chen C.-K., Johnston J.B., Hopkins S.D., Leung S.S.F., Jacobson M.P., Engel J.C., McKerrow J.H., Podust L.M. (2010). A Nonazole CYP51 Inhibitor Cures Chagas’ Disease in a Mouse Model of Acute Infection. Antimicrob. Agents Chemother..

[21] Jiang C., Huang H., Kang X., Yang L., Xi Z., Sun H., Pluth M.D., Yi L. (2021). NBD-Based Synthetic Probes for Sensing Small Molecules and Proteins: Design, Sensing Mechanisms and Biological Applications. Chem. Soc. Rev..

[22] Jagu E., Pomel S., Pethe S., Cintrat J.-C., Loiseau P.M., Labruère R. (2019). Spermine-NBD as Fluorescent Probe for Studies of the Polyamine Transport System in Leishmania Donovani. Bioorg. Med. Chem. Lett..

[23] Martín-Escolano R., Molina-Carreño D., Martín-Escolano J., Clares M.P., Galiana-Roselló C., González-García J., Cirauqui N., Llinares J.M., Rosales M.J., García-España E. (2023). Identification of Aryl Polyamines Derivatives as Anti-Trypanosoma Cruzi Agents Targeting Iron Superoxide Dismutase. Pharmaceutics.

[24] Veiga-Santos P., Li K., Lameira L., de Carvalho T.M.U., Huang G., Galizzi M., Shang N., Li Q., Gonzalez-Pacanowska D., Hernandez-Rodriguez V. (2015). SQ109, a New Drug Lead for Chagas Disease. Antimicrob. Agents Chemother..

[25] Manzano J.I., Cueto-Díaz E.J., Olías-Molero A.I., Perea A., Herraiz T., Torrado J.J., Alunda J.M., Gamarro F., Dardonville C. (2019). Discovery and Pharmacological Studies of 4-Hydroxyphenyl-Derived Phosphonium Salts Active in a Mouse Model of Visceral Leishmaniasis. J. Med. Chem..

[26] Camargo E. (1964). Growth and Differentiation in Trypanosoma Cruzi. I. Origin of Metacyclic Trypanosomes in Liquid Media. Rev. Inst. Med. São Paulo.

[27] Cupello M.P., de Souza C.F., Buchensky C., Soares J.B.R.C., Laranja G.A.T., Coelho M.G.P., Cricco J.A., Paes M.C. (2011). The Heme Uptake Process in Trypanosoma Cruzi Epimastigotes Is Inhibited by Heme Analogues and by Inhibitors of ABC Transporters. Acta Trop..

