# Supplementary material for: Mechanistic Exploration of N,N′-Disubstituted Diamines as Promising Chagas Disease Treatments
Source: Pharmaceuticals (Basel). 2026 Jan 9;19(1):119. doi: 10.3390/ph19010119 (PMC12845068; doi:10.3390/ph19010119)
Supplement: Supplementary file 1 [file pharmaceuticals-19-00119-s001.zip › pharmaceuticals-3929252-supplementary.pdf]

Mechanistic Exploration of *N,N´*-Disubstituted Diamines as Promising Chagas Disease Treatments

Alejandro I. Recio-Balsells,[1] Chantal Reigada,[2,3] Esteban Panozzo-Zénere,[1] María Gabriela Mediavilla,[4] Miguel Villarreal-Parra,[1] María Patricia Doyle,[5] Juan C. Engel,[5] Julia A. Cricco,[4] Claudio A. Pereira[2,3] and Guillermo R. Labadie[1,6,*]

[1]Instituto de Química Rosario (IQUIR-CONICET-UNR), Suipacha 531 S2002LRK, Rosario, Argentina
[2]Universidad de Buenos Aires, Facultad de Medicina, Instituto de Investigaciones Médicas A. Lanari, Buenos Aires, Argentina
[3]Consejo Nacional de Investigaciones Científicas y Técnicas, Universidad de Buenos Aires, Instituto de Investigaciones Médicas (IDIM), Laboratorio de Parasitología Molecular, Buenos Aires, Argentina
[4]Instituto de Biología Molecular y Celular de Rosario (IBR-CONICET-UNR), Ocampo y Esmeralda, 2000, Rosario, Argentina
[5]Sandler Center for Drug Discovery, University of California San Francisco, San Francisco, California, United States of America, Department of Pathology, University of California San Francisco, San Francisco, California, United States of America
[6]Departamento de Química Orgánica, Facultad de Ciencias Bioquímicas y Farmacéuticas, Universidad Nacional de Rosario, Suipacha 531 S2002LRK, Rosario, Argentina

Supporting Information

Contents

1. Compound´s SMILES. 2
2. Compound´s LE and LLE 3
3. NMR spectra and ESI HRMS **INT 1** and **4c-NBD**. 4

1. Compound´s SMILES.

Table S1. Compound´s SMILES.

ID	n	R	SMILES
**3a**	3	4-H	C(CNCc1ccccc1)CNCc1ccccc1
**4a**	4	4-H	C(CNCc1ccccc1)CCNCc1ccccc1
**6a**	6	4-H	C(CCNCc1ccccc1)CCCNCc1ccccc1
**8a**	8	4-H	C(CCCNCc1ccccc1)CCCCNCc1ccccc1
**10a**	10	4-H	C(CCCCNCc1ccccc1)CCCCCNCc1ccccc1
**12a**	12	4-H	C(CCCCCNCc1ccccc1)CCCCCCNCc1ccccc1
**3b**	3	4-OMe	COc1ccc(cc1)CNCCCNCc1ccc(cc1)OC
**4b**	4	4-OMe	COc1ccc(cc1)CNCCCCNCc1ccc(cc1)OC
**6b**	6	4-OMe	COc1ccc(cc1)CNCCCCCCNCc1ccc(cc1)OC
**8b**	8	4-OMe	COc1ccc(cc1)CNCCCCCCCCNCc1ccc(cc1)OC
**10b**	10	4-OMe	COc1ccc(cc1)CNCCCCCCCCCCNCc1ccc(cc1)OC
**12b**	12	4-OMe	COc1ccc(cc1)CNCCCCCCCCCCCCNCc1ccc(cc1)OC
**3c**	3	4-OBn	C(CNCc1ccc(cc1)OCc1ccccc1)CNCc1ccc(cc1)OCc1ccccc1
**4c**	4	4-OBn	C(CNCc1ccc(cc1)OCc1ccccc1)CCNCc1ccc(cc1)OCc1ccccc1
**6c**	6	4-OBn	C(CCNCc1ccc(cc1)OCc1ccccc1)CCCNCc1ccc(cc1)OCc1ccccc1
**8c**	8	4-OBn	C(CCCNCc1ccc(cc1)OCc1ccccc1)CCCCNCc1ccc(cc1)OCc1ccccc1
**10c**	10	4-OBn	C(CCCCNCc1ccc(cc1)OCc1ccccc1)CCCCCNCc1ccc(cc1)OCc1ccccc1
**12c**	12	4-OBn	C(CCCCCNCc1ccc(cc1)OCc1ccccc1)CCCCCCNCc1ccc(cc1)OCc1ccccc1
**3d**	3	3-OH 4-OMe	COc1ccc(cc1O)CNCCCNCc1ccc(c(c1)O)OC
**4d**	4	3-OH 4-OMe	COc1ccc(cc1O)CNCCCCNCc1ccc(c(c1)O)OC
**6d**	6	3-OH 4-OMe	COc1ccc(cc1O)CNCCCCCCNCc1ccc(c(c1)O)OC
**8d**	8	3-OH 4-OMe	COc1ccc(cc1O)CNCCCCCCCCNCc1ccc(c(c1)O)OC
**10d**	10	3-OH 4-OMe	COc1ccc(cc1O)CNCCCCCCCCCCNCc1ccc(c(c1)O)OC
**12d**	12	3-OH 4-OMe	COc1ccc(cc1O)CNCCCCCCCCCCCCNCc1ccc(c(c1)O)OC
**3e**	3	3-OMe 4-OBn	COc1cc(CNCCCNCc2ccc(c(c2)OC)OCc2ccccc2)ccc1OCc1ccccc1
**4e**	4	3-OMe 4-OBn	COc1cc(CNCCCCNCc2ccc(c(c2)OC)OCc2ccccc2)ccc1OCc1ccccc1
**6e**	6	3-OMe 4-OBn	COc1cc(CNCCCCCCNCc2ccc(c(c2)OC)OCc2ccccc2)ccc1OCc1ccccc1
**8e**	8	3-OMe 4-OBn	COc1cc(CNCCCCCCCCNCc2ccc(c(c2)OC)OCc2ccccc2)ccc1OCc1ccccc1
**10e**	10	3-OMe 4-OBn	COc1cc(CNCCCCCCCCCCNCc2ccc(c(c2)OC)OCc2ccccc2)ccc1OCc1cccc c1
**12e**	12	3-OMe 4-OBn	COc1cc(CNCCCCCCCCCCCCNCc2ccc(c(c2)OC)OCc2ccccc2)ccc1OCc1c cccc1

2. Compound´s LE and LLE

Table S2. Values of compound's ligand efficiency (LE) and ligand lipophilic efficiency (LLE).

ID	n	R	LE	LLE
**3a**	3	Benzyl	0.43323	3.6782
**4a**	4	Benzyl	0.32232	1.9228
**6a**	6	Benzyl	0.29302	1.014
**8a**	8	Benzyl	0.27746	0.26007
**10a**	10	Benzyl	0.24794	-0.80363
**12a**	12	Benzyl	0.23023	-1.7124
**3b**	3	4-Methoxy	0.28028	2.5172
**4b**	4	4-Methoxy	0.2686	2.0628
**6b**	6	4-Methoxy	0.35347	3.154
**8b**	8	4-Methoxy	-	-
**10b**	10	4-Methoxy	0.21488	-0.66363
**12b**	12	4-Methoxy	0.20145	-1.5724
**3c**	3	4-Benzyloxy	0.22945	0.83587
**4c**	4	4-Benzyloxy	0.23408	0.67027
**6c**	6	4-Benzyloxy	0.23263	0.062499
**8c**	8	4-Benzyloxy	0.16877	-2.3692
**10c**	10	4-Benzyloxy	0.15855	-3.3449
**12c**	12	4-Benzyloxy	0.14651	-4.4086
**3d**	3	3-OH,4OMe	0.25786	3.2086
**4d**	4	3-OH,4OMe	0.24794	2.7542
**6d**	6	3-OH,4OMe	0.26922	2.6412
**8d**	8	3-OH,4OMe	0.21488	0.93657
**10d**	10	3-OH,4OMe	0.20947	0.21486
**12d**	12	3-OH,4OMe	0.1896	-0.88103
**3e**	3	3-OMe.4-OBn	0.23726	1.8667
**4e**	4	3-OMe.4-OBn	0.17305	-0.28664
**6e**	6	3-OMe.4-OBn	0.15349	-1.5422
**8e**	8	3-OMe.4-OBn	0.14651	-2.451
**10e**	10	3-OMe.4-OBn	0.14014	-3.3598
**12e**	12	3-OMe.4-OBn	0.1343	-4.2686

3. NMR spectra of fluorescent analog and intermediates.

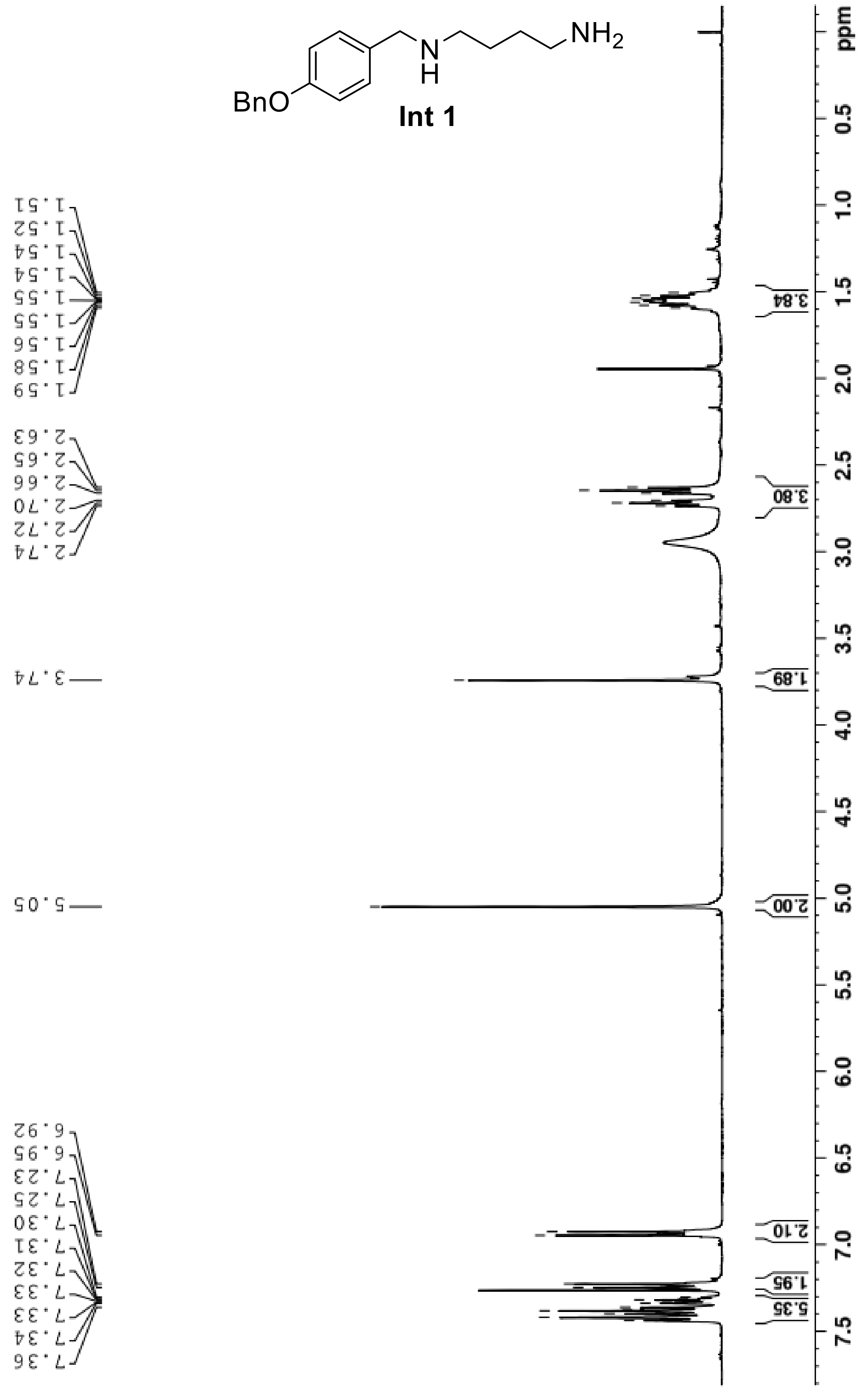

Figure S1. ^{1}H NMR of N^1-(4-(benzyloxy)benzyl)butane-1,4-diamine (**Int 1**)

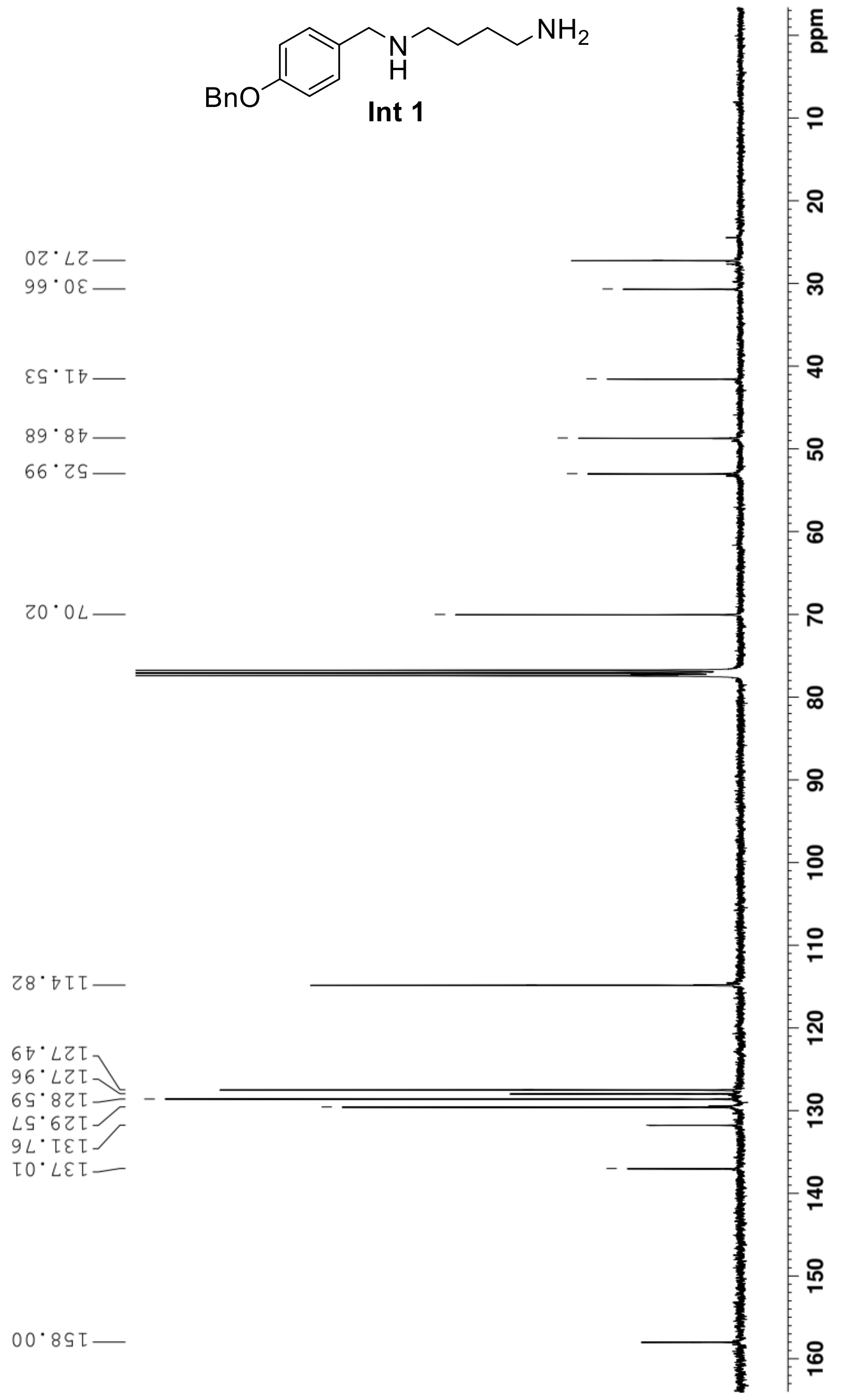

Figure S2. ^{13}C NMR of N[1]-(4-(benzyloxy)benzyl)butane-1,4-diamine (**Int 1**)

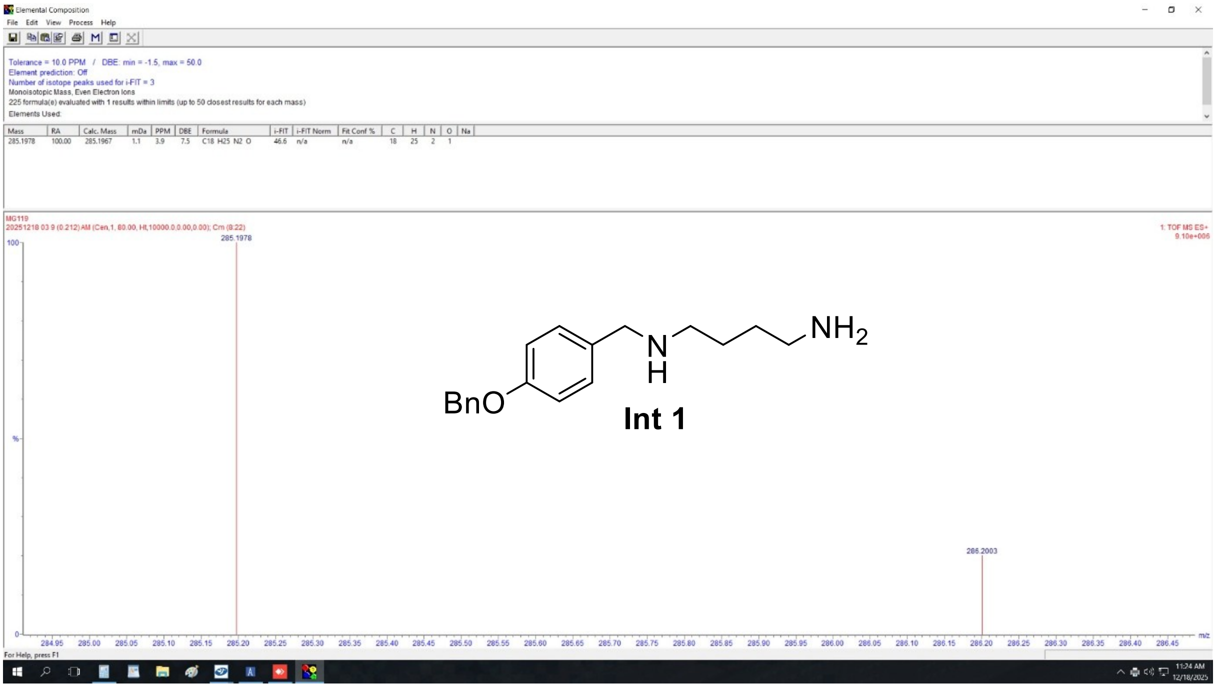

Tolerance = 10.0 PPM / DBE: min = -1.5, max = 50.0
Element prediction: Off
Number of isotope peaks used for i-FIT = 3

Monoisotopic Mass, Even Electron Ions
225 formula(e) evaluated with 1 results within limits (up to 50 closest results for each mass)
Elements Used:
C: 18-24 H: 20-26 N: 0-200 O: 1-8 Na: 0-1
MG119
20251218 03 9 (0.212) AM (Cen,1, 80.00, Ht,10000.0,0.00,0.00); Cm (8:22)   1: TOF MS ES+ 9.10e+006

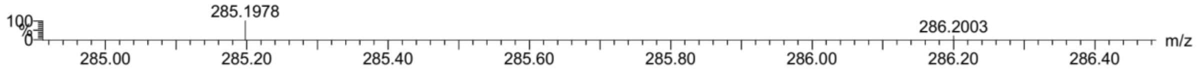

Minimum:	80.00				-1.5				
Maximum:	100.00		5.0	10.0	50.0				
Mass	RA	Calc. Mass	mDa	PPM	DBE	i-FIT	Norm	Conf(%)	Formula
285.1978	100.00	285.1967	1.1	3.9	7.5	46.6	n/a	n/a	C18 H25 N2 O

Figure S3. ESI-HRMS of N[1]-(4-(benzyloxy)benzyl)butane-1,4-diamine (**Int 1**)

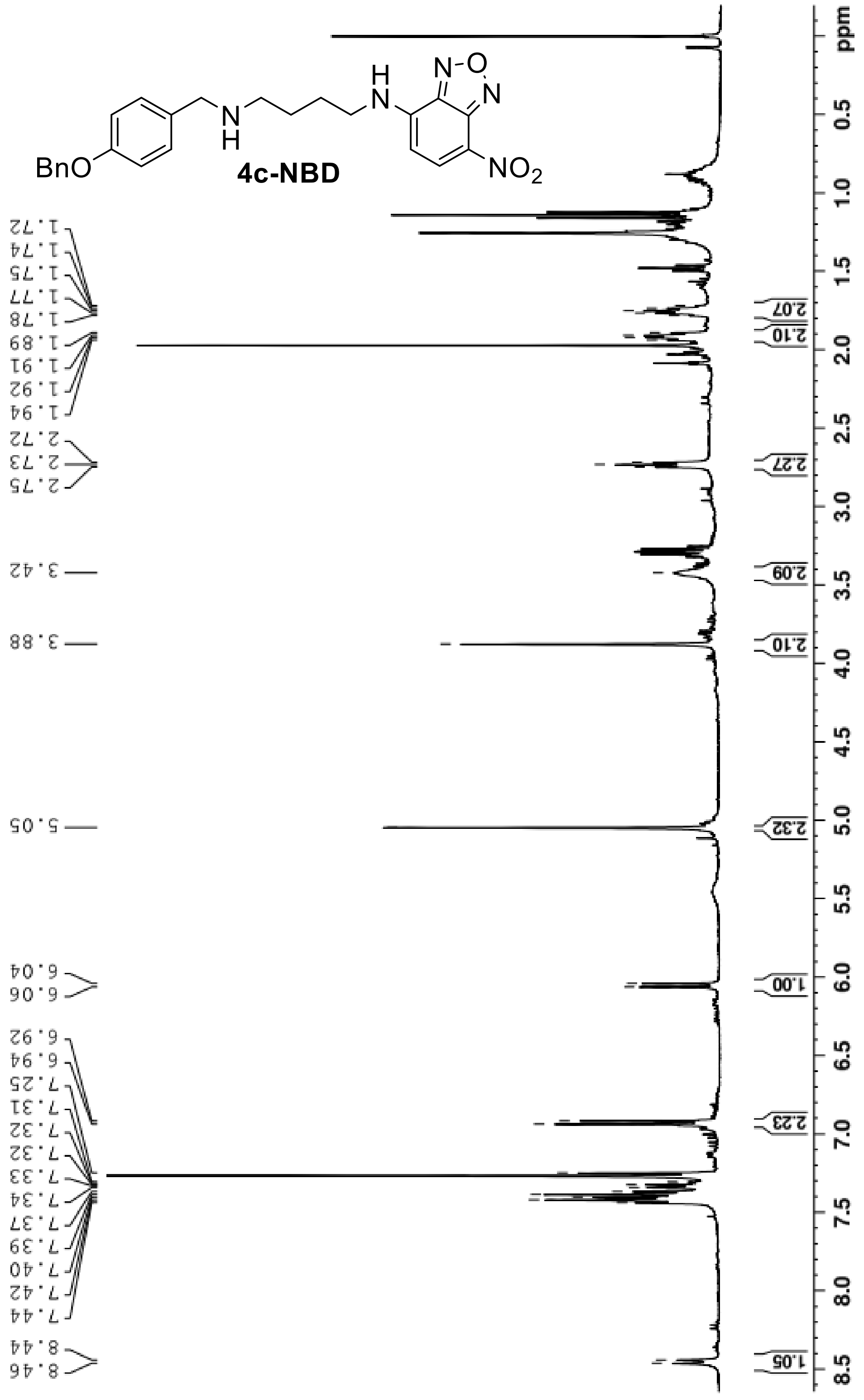

Figure S4. ¹H NMR of *N*-(4-(4-(benzyloxy)benzylamino)butyl)-7-nitrobenzo[c][1,2,5]oxadiazol-4-amine (**4c-NBD)**

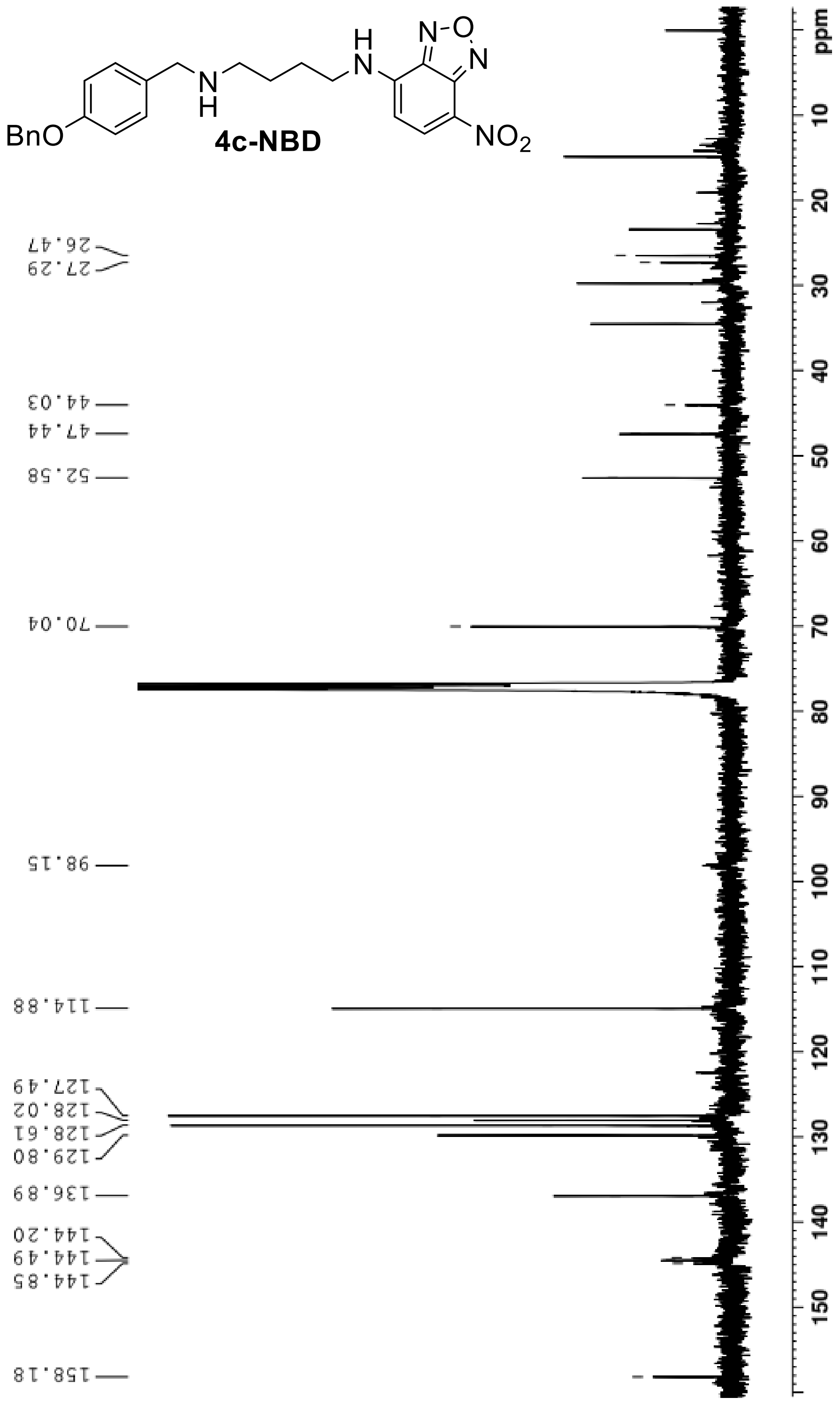

Figure S5. ^{13}C NMR of *N*-(4-(4-(benzyloxy)benzylamino)butyl)-7-nitrobenzo[c][1,2,5]oxadiazol-4-amine (**4c-NBD)**

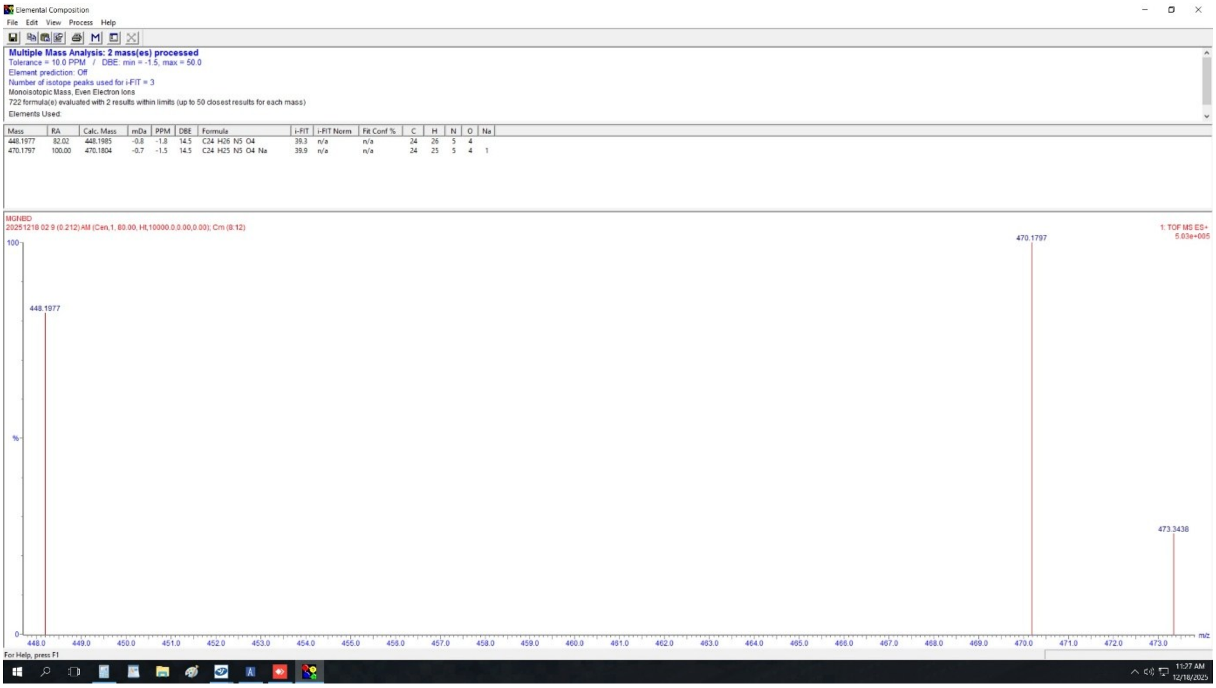

Multiple Mass Analysis: 2 mass(es) processed
Tolerance = 10.0 PPM / DBE: min = -1.5, max = 50.0
Element prediction: Off
Number of isotope peaks used for i-FIT = 3

Monoisotopic Mass, Even Electron Ions
722 formula(e) evaluated with 2 results within limits (up to 50 closest results for each mass)
Elements Used:
C: 24-24 H: 20-26 N: 0-200 O: 1-8 Na: 0-1

MGNBD
20251218 02 9 (0.212) AM (Cen,1, 80.00, Ht,10000.0,0.00,0.00); Cm (8:12)

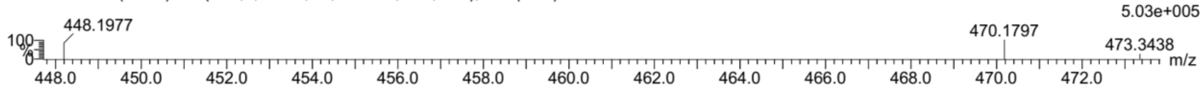

Minimum:	80.00				-1.5				
Maximum:	100.00		5.0	10.0	50.0				

Mass	RA	Calc. Mass	mDa	PPM	DBE	i-FIT	Norm	Conf(%)	Formula
448.1977	82.02	448.1985	-0.8	-1.8	14.5	39.3	n/a	n/a	C24 H26 N5 O4
470.1797	100.00	470.1804	-0.7	-1.5	14.5	39.9	n/a	n/a	C24 H25 N5 O4 Na

Figure S6. ESI-HRMS of *N*-(4-(4-(benzyloxy)benzylamino)butyl)-7-nitrobenzo[c][1,2,5]oxadiazol-4-amine (**4c-NBD)**
